# Dietary patterns and decreased muscle strength incidence: findings from the Korean Genome and Epidemiology Study

**DOI:** 10.1016/j.jnha.2026.100802

**Published:** 2026-02-08

**Authors:** Yuji Jeong, Seok-Won Son, Se-Hong Kim, Ha-Na Kim

**Affiliations:** Department of Family Medicine, St. Vincent’s Hospital, College of Medicine, The Catholic University of Korea, Seoul, Republic of Korea

**Keywords:** Handgrip strength, Decreased muscle strength, Dietary patterns, Macronutrients

## Abstract

**Objectives:**

Muscle strength is a fundamental determinant of functional capacity across adulthood. While dietary protein has been widely studied, prospective evidence considering overall dietary patterns remains inconsistent. We examined the association between macronutrient-based dietary patterns and incident decreased muscle strength among adults aged 40 years and older.

**Methods:**

We analyzed 31,968 adults aged ≥40 years without decreased muscle strength at baseline from the Korean Genome and Epidemiology Study. The participants were categorized into four groups according to macronutrient energy proportions: high-carbohydrate, high-fat, high-protein, and normal diets. The incidence of decreased muscle strength was defined as <28 kg in men and <18 kg in women at follow-up. Cox proportional hazards models estimated hazard ratios (HRs) and 95% confidence intervals (CIs) of decreased muscle strength between groups, and linear mixed-effects models were used to evaluate longitudinal changes in handgrip strength, including time, group, and the group-by-time interaction.

**Results:**

During a median follow-up of 4.0 years, 2,194 participants developed incident decreased muscle strength (incidence rate 1.65 per 100 person-years). The high-protein dietary pattern was associated with a higher risk of incident decreased muscle strength compared with the normal diet (Adjusted HR 1.45, 95% CI 1.06−1.99), whereas no significant associations were observed for high-carbohydrate or high-fat dietary patterns. Changes in dietary patterns from baseline to follow-up were not significantly associated with the risk of decreased muscle strength, and the magnitude of change in handgrip strength over time did not differ across dietary pattern groups.

**Conclusions:**

In middle-aged and older Korean adults, the high-protein dietary pattern was associated with an increased risk of incident muscle strength decline compared to the normal dietary pattern, whereas high-carbohydrate and high-fat patterns were not. These findings suggest that a balanced macronutrient composition, rather than protein intake alone, may be relevant to muscle strength.

## 1 Introduction

Muscle strength is a core component of muscle health and a key determinant of functional capacity throughout adulthood [1]. Reduced muscle strength is independently associated with adverse outcomes, including mobility limitation, disability, and mortality [[2], [3], [4]]. Population-based studies have shown that handgrip strength follows a distinct life-course pattern, peaking in early adulthood and remaining relatively stable through early midlife before declining thereafter [5]. Consistent with this life-course perspective, recent expert consensus has emphasized muscle strength as a primary target for muscle health [6]. The 2025 Asian Working Group for Sarcopenia update extends this focus beyond older adults to include middle-aged populations [7]. Notably, lower muscle strength in midlife has been associated with subsequent adverse health outcomes in later life [8], underscoring the need for preventive strategies that target muscle strength before the onset of a disability.

Nutrition is a potentially modifiable determinant of muscle health across the adult life course [9]. Previous studies have shown that nutritional interventions, particularly those targeting adequate protein intake, may improve muscle mass, muscle strength, and physical performance [10,11]. In observational and longitudinal studies, dietary protein intake has been the most examined nutritional factor in relation to muscle strength, with several studies suggesting that higher protein intake may attenuate declines in handgrip strength over time [12,13]. Notably, a prospective study conducted in a Korean cohort of middle-aged and older adults examined the association between dietary protein intake and the incidence of decreased muscle strength, reporting mixed findings across protein intake levels [14].

Beyond individual nutrients, dietary patterns provide a more comprehensive approach to habitual dietary intakes. Dietary pattern analysis reflects the combined and interactive effects of macronutrients consumed in relative proportions, which may better capture real-world dietary exposures than single-nutrient approaches [15]. Recent studies have suggested that diet quality, anti-inflammatory dietary patterns, and consumption of ultra-processed foods may be associated with muscle strength and longitudinal changes in muscle strength [16,17]. However, much of this evidence has been derived from cross-sectional analyses or has focused on changes in muscle strength over time rather than on the development of new-onset decreased muscle strength.

Therefore, this study aimed to investigate the association between macronutrient-based dietary patterns and the incidence of decreased muscle strength among adults aged 40 years and older using a large community-based prospective cohort.

## 2 Material and methods

### 2.1 Study design and participants

This study utilized data from the Korean Genome and Epidemiology Study (KoGES), a longitudinal cohort study started in 2004, targeting Korean adults aged 40 and above, and managed by the Korea Disease Control and Prevention Agency. The main objective of the KoGES was to collect information on lifestyle factors and various biomarkers to investigate the effects of genes, clinical markers, and the environment and their interactions on the incidence of chronic diseases [18]. The initial cohort comprised individuals who underwent health assessments at a health promotion center from 2004 to 2013, with follow-up assessments occurring between 2012 and 2016.

The KoGES protocol received approval from the Institutional Review Board of the Korean Center for Disease Control and Prevention, and all participants gave written informed consent. The data were anonymized to ensure no personal information was included in the study. The Institutional Review Board of the Catholic University of Korea approved this study (approval number: VC24ZISI0150), which adhered to the principles of the Declaration of Helsinki.

**Fig. 1:**
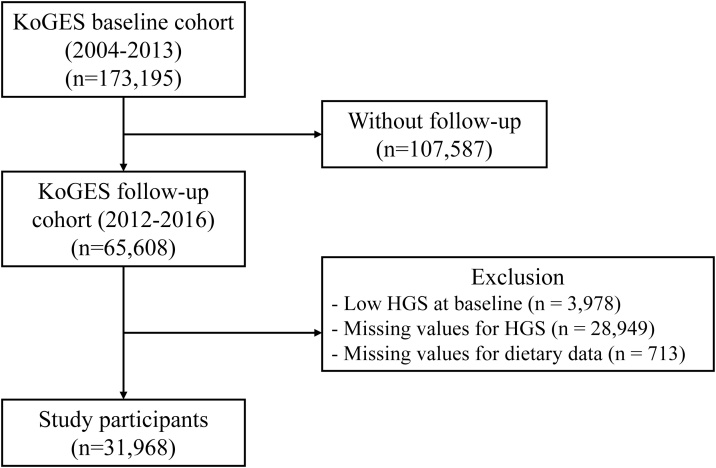
Study participant flow: Data from the Korea Genome Epidemiology Study. Abbreviations: KoGES, the Korean Genome and Epidemiology Study; HGS, hand grip strength.

Out of the 173,195 initial KoGES participants, those not part of the follow-up survey (n = 107,587), those with missing data or values for key variables (n = 29,662), and those with low muscle strength at enrollment (n = 3,978) were excluded. Consequently, 31,968 participants (10,995 men and 20,973 women) were included in the study, and they were categorized into four groups based on the food frequency questionnaire (FFQ) according to the energy proportions from carbohydrates, proteins, or fats: high-carbohydrate diet (HCHO), high-fat diet (HF), high-protein diet (HP), and normal diet (ND) (Fig. 1).

### 2.2 Dietary assessments

Trained dietitians assessed the participants' protein intake using a 103-item semi-quantitative FFQ [19]. This tool estimated nutrient consumption based on the portion sizes and frequency of each food item consumed over the past year. The daily intake of carbohydrates, proteins, and fats, measured in grams, was calculated by adding up the intake from each food item, using the food composition tables from the Rural Development Administration along with the nutrient database from the Korea Health Industry Development Institute [20]. The energy derived from carbohydrates, fats, and proteins was calculated by applying standard conversion factors to convert grams into kilocalories (4 kcal/g for carbohydrates, 9 kcal/g for fats, and 4 kcal/g for proteins). The energy contribution of each macronutrient was then expressed as a percentage of the total energy intake.

Based on the FFQ data at baseline and follow-up, a diet classified as HCHO consists of carbohydrates providing over 65% of the total energy, with fat and protein each contributing no more than 30% and 20%, respectively. Conversely, an HF diet was characterized by fat supplying more than 30% of the energy, while carbohydrates and protein were restricted to 65% or less and 20% or less, respectively. In an HP diet, protein accounted for more than 20% of the energy intake, whereas carbohydrates and fat were limited to 65% or less and 30% or less, respectively. Individuals in the ND group were those whose dietary intake aligned with the Korean Dietary Reference Intakes, ensuring their consumption included 55–65% carbohydrates, 15–30% fats, and 7–20% proteins.

### 2.3 Incidence of decreased muscle strength

Handgrip strength (HGS) was used as an indicator of skeletal muscle strength. Trained examiners assessed the participants' HGS using a digital grip strength dynamometer (KS-301, Lavisen, Seoul, South Korea) during the initial and follow-up surveys. This device can measure forces of up to 90.0 kg and features an adjustable grip span. The participants were instructed to sit with their elbows bent at a 90 ° angle, look straight ahead, and squeeze the dynamometer with maximum effort until a reading appeared on the liquid-crystal display. HGS was measured once for each hand, beginning with the dominant hand. The highest value from the two measurements was used for the HGS analysis. Decreased muscle strength was defined as HGS <28 kg for men and <18 kg for women during follow-up [21].

### 2.4 Covariates

Participants provided self-reported information regarding their sex, age, household income, education level, smoking habits, alcohol consumption, and physical activity. Smoking and drinking statuses were categorized as non-smokers/drinkers, former smokers/drinkers, and current smokers/drinkers. Those who answered "yes" to the question, "Do you regularly exercise to the point of sweating?" were placed in the regular-exercise category. Household income was calculated by dividing the total monthly household income by the square root of the number of household members, and it was split into two groups: those earning 30% above or below the median. The classification of educational levels is as follows: ≤6 years: elementary school or less, 7–12 years: middle or high school, and ≥13 years: college or higher. the presence of comorbidities (hypertension, diabetes, dyslipidemia, cardiovascular disease, stroke, cancer, and chronic obstructive pulmonary disease) were defined as physician-diagnosed conditions reported at baseline or identified before the occurrence of incident decreased muscle strength. Body weight, waist circumference, and height were measured with the participants wearing light indoor clothing without shoes. The body mass index (BMI) was calculated as weight (kg) divided by height squared (m^2^).

### 2.5 Statistical analyses

We used t-tests to compare continuous variables and chi-square tests to compare dichotomous variables. The data are presented as medians with interquartile ranges or as numbers with percentages. To explore the associations between macronutrient-based dietary patterns and the risk of decreased muscle strength, we utilized Cox proportional hazards models. We used an unadjusted model along with three adjusted models. Model 1 accounted for age, sex, and baseline HGS. Model 2 further included BMI and total energy intake. Model 3 was adjusted for smoking status, alcohol consumption, physical activity, education level, household income, and comorbidities. The covariates included BMI, total energy intake, smoking status, alcohol consumption, and physical activity, which were treated as time-varying covariates, whereas age, sex, baseline HGS, education level, household income, and comorbidities, which were treated as fixed covariates. The Kaplan-Meier method was used to evaluate the cumulative incidence of decreased muscle strength across groups, and log-rank tests were conducted. Adjusted incidence curves from the Cox proportional hazard models were used to examine differences in cumulative incidence of decreased muscle strength between groups, accounting for the aforementioned covariates. Restricted cubic spline models, adjusted for all covariates, were used to investigate the relationship between macronutrient intake proportions and the risk of decreased muscle strength. Linear mixed-effects models with a random intercept were used with time, group, and group-by-time interaction as fixed effects to evaluate longitudinal changes in HGS, adjusting for all covariates. All statistical analyses were conducted using SAS version 9.4 (SAS Institute, Cary, NC, USA), with statistical significance determined at p < 0.05.

## 3 Results

### 3.1 Characteristics of the study population by dietary patterns

This study included 31,968 participants (10,995 men and 20,973 women) with a median age of 54.0 years, and 86.8% were classified into the high-carbohydrate diet group. Age differed significantly across dietary patterns (p < 0.001); participants in the ND and HF groups were younger, whereas those in the HP group were older. Sex distribution was similar across groups (p = 0.077). Smoking status, alcohol consumption, and regular exercise differed by dietary pattern, as did household income and educational level (all p < 0.05).

Body mass index differed across dietary patterns (p = 0.009), whereas waist circumference did not show a significant difference (p = 0.496). The prevalence of diabetes, hypertension, dyslipidemia, stroke, and coronary artery disease varied across groups, while chronic obstructive pulmonary disease and cancer did not. Total energy intake differed significantly across dietary patterns (p < 0.001), with higher energy intake observed in the HF and HP groups. Absolute intakes of carbohydrate, fat, and protein, as well as the proportion of energy derived from each macronutrient, differed significantly among groups (all p < 0.001). Median handgrip strength also differed significantly among dietary patterns (p = 0.005) (Table 1).

**Table 1:** Characteristics of the study participants.

	Total	ND	HCHO	HF	HP	p
N	31,968	3,709	27,734	105	420	
Age, years	54.0 (48.0−60.0)	51.0 (45.0−57.0)	54.0 (48.0−60.0)	50.0 (46.0−56.0)	55.0 (50.0−61.0)	<0.001
Sex, n (%)						0.077
Male	10,995 (34.4)	1,338 (36.1)	9,471 (34.1)	42 (40.0)	144 (34.3)	
Female	20,973 (65.6)	2,371 (63.9)	18,263 (65.9)	63 (60.0)	276 (65.7)	
Smoking, n (%)						<0.001
None	23,139 (72.6)	2,535 (68.8)	20,243 (73.2)	71 (67.6)	290 (69.4)	
Ex-smoker	5,340 (16.8)	629 (17.1)	4,608 (16.7)	24 (22.9)	79 (18.9)	
Current smoker	3,383 (10.6)	521 (14.1)	2,803 (10.1)	10 (9.5)	49 (11.7)	
Alcohol consumption, n (%)						<0.001
None	16,051 (50.4)	1,584 (43.0)	14,250 (51.6)	42 (40.0)	175 (42.0)	
Ex-drinker	1,307 (4.1)	141 (3.8)	1,135 (4.1)	7 (6.7)	24 (5.8)	
Current drinker	14,468 (45.5)	1,955 (53.1)	12,239 (44.3)	56 (53.3)	218 (52.3)	
Regular exercise, n (%)						0.016
No	13,654 (42.8)	1,558 (42.2)	11,897 (43.0)	49 (46.7)	150 (35.7)	
Yes	18,229 (57.2)	2,138 (57.8)	15,765 (57.0)	56 (53.3)	270 (64.3)	
Household income, low, n (%)	3,215 (10.4)	243 (6.7)	2,914 (10.8)	7 (6.9)	51 (12.4)	<0.001
Education level, n (%)						<0.001
Elementary	582 (1.8)	30 (0.8)	541 (2.0)	0 (0.0)	11 (2.6)	
Middle or high	8,790 (27.7)	649 (17.6)	8,038 (29.2)	22 (21.0)	81 (19.4)	
≥College	22,391 (70.5)	3,001 (81.5)	18,982 (68.9)	83 (79.0)	325 (77.9)	
Comorbidity, n (%)						
Diabetes Mellitus	2,191 (6.9)	202 (5.5)	1,949 (7.0)	5 (4.8)	35 (8.3)	0.002
Hypertension	6,544 (20.5)	598 (16.1)	5,843 (21.1)	23 (21.9)	80 (19.0)	<0.001
Dyslipidemia	3,720 (11.6)	356 (9.6)	3,308 (11.9)	15 (14.3)	41 (9.8)	<0.001
Stroke	348 (1.1)	19 (0.5)	325 (1.2)	1 (1.0)	3 (0.7)	0.001
Coronary artery disease	913 (2.9)	79 (2.1)	821 (3.0)	3 (2.9)	10 (2.4)	0.037
COPD	56 (0.2)	6 (0.2)	47 (0.2)	0 (0.0)	3 (0.7)	0.118
Cancer	1,300 (4.1)	133 (3.6)	1,142 (4.1)	2 (1.9)	23 (5.5)	0.124
Weight, kg	60.9 (54.9−68.2)	61.2 (55.0−69.2)	60.8 (54.8−68.0)	60.7 (54.0−72.0)	61.6 (55.7−69.6)	0.002
Height, cm	160.0 (155.0−166.2)	161.0 (156.0−167.2)	160.0 (155.0−166.0)	161.0 (156.0−165.6)	159.2 (154.7−166.1)	<0.001
Waist circumference, cm	80.2 (74.0−86.5)	80.0 (74.0−87.0)	80.2 (74.0−86.4)	78.3 (74.0−89.0)	81.0 (75.0−87.7)	0.496
Body mass index, kg/m^2^	23.7 (21.9−25.7)	23.7 (21.8−25.7)	23.7 (21.9−25.7)	23.9 (21.6−26.1)	24.1 (22.4−26.2)	0.009
Energy intake, kcal/day	1666.9 (1396.2−1987.6)	1868.2 (1454.0−2332.2)	1650.3 (1392.8−1942.2)	1974.9 (1264.2−2575.7)	1825.6 (1309.1−2354.8)	<0.001
Carbohydrate intake, g/day	307.9 (259.4−356.3)	288.1 (222.9−358.5)	309.8 (265.7−356.2)	235.2 (151.5−327.3)	250.9 (179.3−335.2)	<0.001
Fat intake, g/day	24.1 (16.7−34.0)	45.0 (34.5−56.6)	22.3 (15.7−30.4)	72.1 (44.0−88.0)	44.5 (30.6−60.6)	<0.001
Protein intake, g/day	54.3 (43.1−68.5)	76.8 (59.8−95.9)	52.3 (42.0−64.5)	82.8 (53.5−112.7)	97.5 (70.7−125.7)	<0.001
Proportion of energy intake, %						
Carbohydrate	73.5 (68.5−77.8)	62.2 (59.8−63.8)	74.6 (70.7−78.4)	50.4 (47.7−52.3)	55.6 (52.3−58.8)	<0.001
Fat	13.3 (10.1−16.9)	21.6 (20.1−23.5)	12.5 (9.7−15.3)	31.6 (30.6−33.9)	22.8 (19.9−25.9)	<0.001
Protein	13.1 (11.8−14.7)	16.5 (15.5−17.6)	12.8 (11.6−14.0)	17.7 (16.3−18.7)	21.2 (20.5−22.6)	<0.001
Hand grip strength, kg	27.1 (23.0−35.8)	27.7 (23.1−36.5)	27.1 (23.0−35.7)	28.3 (24.1−36.4)	26.4 (22.6−33.9)	0.005
Incidence of decreased muscle strength, n (%)	2,194 (6.9)	207 (5.6)	1935 (7.0)	8 (7.6)	44 (10.5)	<0.001

Data are presented as n (%) or median (interquartile range). Abbreviation; COPD, Chronic obstructive pulmonary disease; HCHO, high-carbohydrate diet; HF, high-fat diet; HP, high-protein diet; ND, normal diet.

### 3.2 Cumulative impacts of dietary patterns on the incidence of decreased muscle strength

**Fig. 2:**
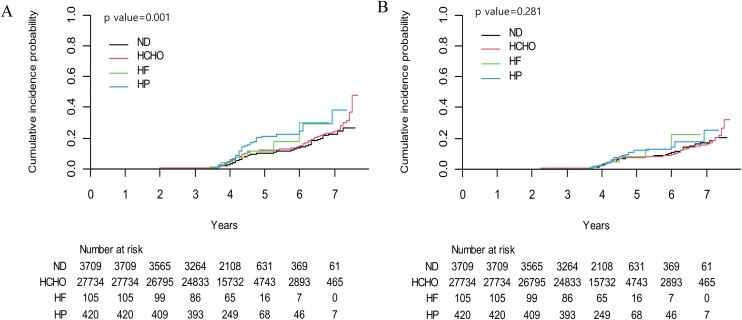
Cumulative incidence of decreased muscle strength according to dietary patterns. A. Unadjusted cumulative incidence curve using the log-rank test. B. Adjusted cumulative incidence curve using the Cox proportional hazard model after adjusting for age, sex, baseline muscle strength, body mass index, total energy intake, smoking status, alcohol consumption, physical activity, educational level, household income, and history of comorbidities. Abbreviations: HCHO, high-carbohydrate diet; HF, high-fat diet; HP, high-protein diet; ND, normal diet.

Fig. 2 shows the cumulative incidence of decreased handgrip strength according to macronutrient-based dietary patterns. The cumulative incidence curves differed significantly across groups (Fig. 2A, p = 0.001). After adjustment for covariates, no significant differences were observed between dietary pattern groups in the cumulative incidence curves of decreased muscle strength (Fig. 2B).

### 3.3 Dietary patterns as a predictor of the incidence of decreased muscle strength

Table 2 shows the associations between macronutrient-based dietary patterns and the risk of decreased muscle strength. Among the 31,968 participants included in the analysis, 2,194 developed low muscle strength during follow-up. The overall incidence rate was 1.65 (95% CI, 1.59–1.73) per 100 person-years, with a median follow-up duration of 4.0 years (interquartile range, 3.8–4.3).

**Table 2:** Association between dietary patterns and the incidence of the decreased muscle strength.

Dietary Patterns	Outcomes	Person-years	Incidence rate^a^	Unadjusted		Model l		Model 2		Model 3	
				HR (95% CI)	p	HR (95% CI)	p	HR (95% CI)	p	HR (95% CI)	p
ND	207/3,709	15297.6	1.35	1		1		1		1	
HCHO	1,935/27,734	11519.0	1.68	1.25 (1.09−1.44)	0.002	1.10 (0.95−1.27)	0.192	0.98 (0.85−1.14)	0.828	0.95 (0.82−1.10)	0.509
HF	8/105	415.5	1.93	1.46 (0.73−2.92)	0.289	1.54 (0.78−3.03)	0.211	1.51 (0.74−3.08)	0.257	1.58 (0.78−3.21)	0.201
HP	44/420	1770.9	2.48	1.84 (1.34−2.54)	<0.001	1.47 (1.09−1.99)	0.012	1.43 (1.05−1.96)	0.024	1.45 (1.06−1.99)	0.020

a Per 100 person-years. Model 1: adjusted for age, sex, and baseline muscle strength; Model 2: adjusted for Model 1 plus body mass index and total energy intake; Model 3: adjusted for Model 2 and smoking status, alcohol consumption, physical activity, household income, education level, and history of comorbidities. Abbreviations: CI, confidence interval; HCHO, high-carbohydrate diet; HF, high-fat diet; HP, high-protein diet; HR, hazard ratio; ND, normal diet.

In unadjusted Cox proportional hazards models, both the HCHO and HP groups were associated with a higher risk of decreased muscle strength compared with the ND group. (unadjusted hazard ratio [HR] 1.25, 95% confidence interval [CI] 1.09–1.44 and HR 1.84, 95% CI, 1.34–2.54, respectively). However, after adjustment for all covariates, the HP diet remained significantly associated with an increased risk of low muscle strength (Adjusted HR 1.45, 95% CI 1.06−1.99).

In the linear mixed-effects models, a significant overall time effect was observed, with handgrip strength decreasing from baseline to follow-up across all dietary pattern groups (p = 0.001). Baseline HGS levels differed significantly among dietary patterns (p < 0.001). However, the group-by-time interaction was not statistically significant (p = 0.372), indicating that the magnitude of change in handgrip strength over time did not differ across dietary pattern groups (Supplementary Table S1).

### 3.4 Change in dietary patterns as a predictor of the incidence of decreased muscle strength

Changes in dietary patterns from baseline to follow-up were evaluated in relation to the incidence of decreased muscle strength (Table 3). In the unadjusted analyses, a transition from the ND to the HCHO dietary pattern was associated with a higher incidence of decreased muscle strength, whereas a transition from the HCHO to the ND pattern was associated with a lower risk of decreased muscle strength. However, after adjustment of all covariates, changes in dietary patterns were not significantly associated with the risk of decreased muscle strength.

**Table 3:** Association between changes in dietary pattern and the incidence of decreased muscle strength.

Dietary pattern		Outcomes	Person-years	Incidence rate^a^	Unadjusted		Model1		Model 2		Model 3	
Baseline	Follow-up				HR (95% CI)	p	HR (95% CI)	p	HR (95% CI)	p	HR (95% CI)	p
ND	ND	42/1,044	4242.6	0.99	1		1		1		1	
	HCHO	151/2,375	9845.7	1.53	1.52 (1.08−2.13)	0.017	1.34 (0.95−1.89)	0.091	1.34 (0.95−1.88)	0.098	1.26 (0.88−1.81)	0.199
	HF	3/86	354.8	1.33	0.96 (0.32−2.86)	0.936	1.21 (0.40−3.62)	0.734	1.23 (0.41−3.70)	0.710	1.20 (0.40−3.61)	0.745
	HP	3/51	217.5	1.38	1.33 (0.45−3.99)	0.606	1.04 (0.35−3.10)	0.950	1.01 (0.34−3.02)	0.992	1.01 (0.33−3.09)	0.981
HCHO	ND	195/3,579	15001.2	1.30	0.71 (0.61−0.82)	<0.001	0.82 (0.71−0.95)	0.008	0.82 (0.71−0.96)	0.011	0.87 (0.75−1.02)	0.089
	HCHO	1693/23,304	96644.5	1.75	1		1		1		1	
	HF	10/171	685.4	1.46	0.86 (0.47−1.58)	0.633	1.14 (0.62−2.09)	0.680	1.15 (0.63−2.11)	0.654	1.14 (0.58−2.23)	0.713
	HP	14/226	954.9	1.47	0.81 (0.49−1.37)	0.437	0.91 (0.54−1.52)	0.717	0.91 (0.54−1.53)	0.725	1.02 (0.61−1.72)	0.934
HF	ND	3/38	142.3	2.11	1.85 (0.07−48.44)	0.712	0.14 (0.00−12.23)	0.385	0.42 (0.01−33.56)	0.696	0.46 (0.00−110.7)	0.782
	HCHO	5/5	232.4	2.15	1.86 (0.07−46.44)	0.705	0.18 (0.00−12.10)	0.427	0.41 (0.01−25.54)	0.675	0.26 (0.00−105.3)	0.662
	HF	0/5	23.7	0.00	1		1		1		1	
	HP	–	–	–	–	–	–	–	–	–	–	–
HP	ND	11/92	396.1	2.78	0.94 (0.23−3.84)	0.936	0.34 (0.08−1.48)	0.151	0.29 (0.06−1.30)	0.106	0.31 (0.06−1.63)	0.167
	HCHO	30/283	1182.7	2.54	0.94 (0.23−3.84)	0.902	0.31 (0.08−1.23)	0.095	0.26 (0.06−1.06)	0.060	0.28 (0.06−1.30)	0.104
	HF	0/3	13.8	0.00	1.14 (0.05−26.11)	0.936	0.33 (0.01−8.41)	0.499	0.30 (0.01−7.95)	0.469	0.24 (0.00−11.55)	0.470
	HP	2/21	91.2	2.19	1		1		1		1	

a Per 100 person-years. Model 1: adjusted for age, sex, and baseline muscle strength; Model 2: adjusted for Model 1 plus body mass index and total energy intake; Model 3: adjusted for Model 2 and smoking status, alcohol consumption, physical activity, household income, education level, and history of comorbidities. Abbreviations: CI, confidence interval; HCHO, high-carbohydrate diet; HF, high-fat diet; HP, high-protein diet; HR, hazard ratio; ND, normal diet.

### 3.5 Subgroup analysis for impacts of dietary patterns on the incidence of decreased muscle strength

Subgroup analyses were conducted according to age, sex, obesity, physical activity, and comorbidity status (Table 4). None of the dietary patterns showed a statistically significant association with the risk of decreased muscle strength in any subgroup when compared with the ND group.

**Table 4:** Association between dietary patterns and the incidence of decreased muscle strength stratified by age, sex, obesity, physical activity, and comorbidities.

	Dietary Patterns	Outcomes	aHR (95% CI)	p	p for interaction
Male	ND	66/1,338	1		0.304
	HCHO	523/9,471	0.92 (0.71−1.21)	0.569	
	HF	2/42	1.17 (0.33−4.15)	0.811	
	HP	15/144	1.66 (0.93−2.95)	0.085	
Female	ND	141/2,371	1		
	HCHO	1412/18,263	1.14 (0.95−1.37)	0.168	
	HF	6/63	1.91 (0.81−4.49)	0.139	
	HP	29/276	1.27 (0.83−1.96)	0.273	
Age <65	ND	162/3,447	1		0.194
	HCHO	1465/24,929	1.15 (0.97−1.36)	0.110	
	HF	7/100	1.57 (0.72−3.45)	0.259	
	HP	33/366	1.41 (0.95−2.10)	0.087	
Age ≥65	ND	45/262	1		
	HCHO	470/2,805	0.91 (0.66−1.26)	0.569	
	HF	1/5	1.57 (0.30−8.21)	0.593	
	HP	11/54	1.43 (0.70−2.91)	0.326	
Comorbidity, no	ND	122/2,677	1		0.622
	HCHO	1080/17,987	1.13 (0.93−1.37)	0.221	
	HF	5/67	1.74 (0.74−4.10)	0.205	
	HP	23/273	1.26 (0.79−2.02)	0.333	
Comorbidity, yes	ND	84/1,029	1		
	HCHO	855/9,734	0.97 (0.77−1.24)	0.833	
	HF	3/38	1.28 (0.36−4.54)	0.700	
	HP	21/147	1.60 (0.96−2.66)	0.071	
Obesity, no	ND	138/2,486	1		0.649
	HCHO	1264/18,679	1.09 (0.90−1.31)	0.375	
	HF	5/66	1.79 (0.76−4.21)	0.181	
	HP	20/258	1.19 (0.74−1.91)	0.480	
Obesity, yes	ND	69/1,223	1		
	HCHO	671/9,055	1.01 (0.78−1.31)	0.933	
	HF	3/39	1.20 (0.34−4.26)	0.783	
	HP	24/162	1.58 (0.95−2.63)	0.080	
Regular exercise, no	ND	94/1,558	1		0.511
	HCHO	967/11,897	1.08 (0.87−1.35)	0.482	
	HF	4/49	1.72 (0.67−4.46)	0.262	
	HP	17/150	1.24 (0.72−2.13)	0.435	
Regular exercise, yes	ND	111/2,138	1		
	HCHO	959/15,765	1.04 (0.85−1.28)	0.687	
	HF	4/56	1.45 (0.50−4.21)	0.496	
	HP	27/270	1.46 (0.93−2.28)	0.101	

Values are expressed as HRs with 95% CIs derived via multivariate Cox proportional hazard models, adjusting for age, sex, body mass index, total energy intake, baseline muscle strength, smoking status, alcohol consumption, physical activity, household income, education level, and history of comorbidities. Abbreviations: aHR, adjusted hazard ratio; CI, confidence interval; HCHO, high-carbohydrate diet; HF, high-fat diet; HP, high-protein diet; ND, normal diet.

### 3.6 Non-linear associations between the proportions of macronutrients intake and incidence of decreased muscle strength

**Fig. 3:**
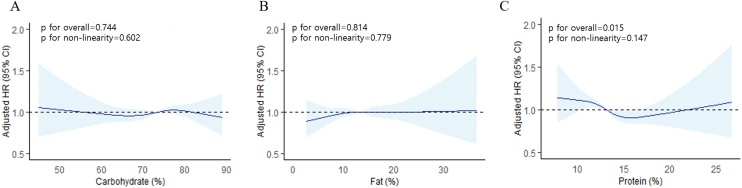
Dose-response analysis of the proportions of macronutrients intake for the risk of decreased muscle strength. *P* values were determined using restricted cubic-spline regression adjusted for age, sex, baseline muscle strength, body mass index, total energy intake, smoking status, alcohol consumption, physical activity, educational level, household income, and history of comorbidities. (A) Energy proportion derived from carbohydrate intake, (B) energy proportion derived from fat intake, and (C) energy proportion derived from protein intake. Abbreviations: HR, hazard ratio; CI, confidence interval.

In nonlinear dose–response analyses using restricted cubic spline models, carbohydrate and fat intake proportions were not significantly associated with the risk of decreased muscle strength (Fig. 3A and B). The proportion of protein intake showed a significant overall association with the risk of decreased muscle strength (p for overall = 0.015), although the nonlinearity was not statistically significant (p for nonlinearity = 0.147) (Fig. 3C).

## 4 Discussion

In this prospective cohort study, which included 31,968 participants aged 40 years and older, we found that macronutrient-based dietary patterns were differentially associated with the incidence of decreased muscle strength. The HP diet showed a higher risk of developing low muscle strength compared with the ND group, even after adjustment for covariates such as age, demographic, lifestyle, and clinical factors. In contrast, HCHO and HF dietary patterns were not significantly associated with low muscle strength after adjustment. These findings suggest that macronutrient balance may provide additional insight into the development of muscle strength impairment from midlife.

Muscle strength represents an integrative marker of neuromuscular function and functional reserve, capturing multiple physiological domains relevant to mobility and survival [22]. Recent studies consistently demonstrate that lower muscle strength is strongly associated with disability, cardiometabolic outcomes, and all-cause mortality [[23], [24], [25]]. Importantly, emerging evidence suggests that muscle strength provides prognostic information beyond structural measures of muscle health, particularly from midlife onward, supporting its use as a clinically meaningful endpoint in population-based research [8,26].

Our results should be interpreted in the context of previous studies examining dietary protein intake and muscle strength. In a Korean prospective cohort of middle-aged and older adults, Kim et al. reported heterogeneous associations between dietary protein intake and the incidence of low muscle strength across different intake levels [14]. Previous studies have reported controversial results regarding the effects of HP diet on muscle strength. Several studies reporting a positive association between protein intake and muscle strength have been conducted in older or frail adult populations, in whom age-related muscle decline and insufficient protein intake are common [[27], [28], [29]], while other studies have reported no positive association between protein intake and muscle strength in different research contexts, including intervention-based studies or disease-specific populations [[30], [31], [32]]. In this context, our findings should be interpreted as reflecting the association between macronutrient-based dietary patterns and the incidence of decreased muscle strength in a generally middle-aged population. Our results are particularly relevant for middle-aged adults, a population increasingly recognized as a critical target for early prevention of functional decline. Recently updated Asian Working Group for Sarcopenia guidelines emphasize the importance of preserving muscle strength before older age. However, nutritional recommendations for this age group have largely focused on increasing protein intake. Our findings suggest that such recommendations should be contextualized within overall dietary patterns and macronutrient balance.

Most previous studies examining the association between protein intake and muscle strength have primarily focused on absolute protein intake. As a result, how protein intake is distributed relative to total energy intake and other macronutrients has been less frequently considered, particularly in relation to functional outcomes such as muscle strength. In this study, protein intake was evaluated in relation to total energy intake, allowing assessment of its proportional contribution within habitual diets. By examining macronutrient intake as relative proportions of total energy, our study adds to existing evidence by examining dietary composition beyond absolute protein intake. While adequate protein intake could be essential for maintaining muscle mass, our findings indicate that a higher proportional intake of protein within the overall diet does not necessarily translate into functional benefits for muscle strength.

This is the first prospective cohort study to investigate the association between dietary patterns and the incidence of decreased muscle strength in Korean adults. However, this study had several limitations. First, the number of participants was uneven across dietary groups, with particularly small sample sizes in the HP and HF diet groups, which may have limited the statistical power to detect significant associations and may be reflected in the broader confidence intervals surrounding the estimates of associations for the outcome. Second, the relatively short follow-up period may have been insufficient to clearly demonstrate the impact of dietary patterns on the prevention or development of decreased muscle strength. Third, macronutrient quality, including the source and composition of macronutrients, was not evaluated, which limited our ability to examine the potential role of dietary quality in relation to muscle strength. Future studies incorporating more detailed dietary characterization and repeated dietary assessments may help clarify the relationship between dietary patterns and muscle strength.

## 5 Conclusions

In this large prospective cohort of Korean adults aged 40 years and older, a HP dietary pattern was associated with an increased risk of incident decreased muscle strength, whereas HCHO and HF patterns were not. These findings support the importance of considering overall dietary balance and macronutrient composition, rather than focusing solely on protein intake, in strategies aimed at preserving muscle strength across the adult life course. Balanced dietary approaches initiated in midlife may be relevant to the maintenance of muscle strength across the adult life course.

## CRediT authorship contribution statement

Yuji Jeong and Ha-Na Kim contributed to the conception and design of the study. Yuji Jeong performed a literature search and drafted the first version of the manuscript. Yuji Jeong, Seok-Won Son, Se-Hong Kim, and Ha-Na Kim analyzed and interpreted the data and provided critical revisions to the manuscript. Ha-Na Kim supervised this study. All authors made significant contributions to finalizing the manuscript and approved the final version for publication.

## Declaration of Generative AI and AI-assisted technologies in the writing process

The authors declare that generative AI and AI-assisted technologies were not used in the writing, analysis, or preparation of this manuscript.

## Funding

This work was supported by the National Research Foundation of Korea (NRF) grant funded by the Korea government (MSIT) (No. RS-2023-00253061).

## Data statement

Data from the Korean Genome and Epidemiology Study supporting the findings of this study are available from the Clinical & Omics Data Archive (CODA, https://coda.nih.go.kr/frt/index.do). Access to the data is restricted; however, they are available upon reasonable request and with permission from CODA.

## Declaration of competing interest

The authors declare that they have no competing financial interests or personal relationships that could have influenced the work reported in this paper.
