# Supplementary material for: Dietary patterns and decreased muscle strength incidence: findings from the Korean Genome and Epidemiology Study
Source: J Nutr Health Aging. 2026 Feb 8;30(4):100802. doi: 10.1016/j.jnha.2026.100802 (PMC12907224; doi:10.1016/j.jnha.2026.100802)
Supplement: Supplementary file 1 [file mmc1.docx]

| Dietary patterns | Baseline | Follow-up | Group effect | p | Time effect | p | Group×time interaction | p |
| --- | --- | --- | --- | --- | --- | --- | --- | --- |
| ND | 29.9(29.8-30.0) | 29.1(29.0-29.2) | Ref. | - | -0.17(-0.22 ‒ -0.11) | <0.001 |  |  |
| HCHO | 29.4(28.2-30.5) | 28.8(27.6-29.9) | 0.36(0.15‒0.56) | 0.001 | -0.20(-0.22 ‒ -0.18) | <0.001 | -0.04(-0.10‒0.02) | 0.198 |
| HF | 29.0(28.4-29.5) | 28.6(28.0-29.1) | -0.19(-1.36‒0.98) | 0.748 | -0.14(-0.47 ‒ 0.19) | 0.392 | 0.02(-0.31‒0.36) | 0.895 |
| HP | 29.6(29.4-29.8) | 28.9(28.7-29.1) | -0.59(-1.20‒0.02) | 0.056 | -0.10(-0.26 ‒ 0.05) | 0.199 | 0.06(-0.11‒0.23) | 0.468 |
| p for overall |  |  |  | <0.001 |  | 0.001 |  | 0.372 |

Supplementary Table 1. Changes in handgrip strength over time according to dietary pattern

Values are presented as β coefficients with 95% confidence intervals. Adjusted for age, sex, body mass index, total energy intake, smoking status, alcohol consumption, physical activity, household income, education level, and history of comorbidities.

Abbreviations: HCHO, high-carbohydrate diet; HF, high-fat diet; HP, high-protein diet; ND, normal diet.
